# Patients' Perceptions of Atrial Fibrillation, Stroke Risk, and Oral Anticoagulation Treatment: An International Survey

**DOI:** 10.1055/s-0038-1666803

**Published:** 2018-07-05

**Authors:** Deirdre A. Lane, Juliane Meyerhoff, Ute Rohner, Gregory Y. H. Lip

**Affiliations:** 1 University of Birmingham Institute of Cardiovascular Sciences, City Hospital, Sandwell and West Birmingham Hospitals NHS Trust, Birmingham, United Kingdom; 2 Aalborg Thrombosis Research Unit, Department of Clinical Medicine, Aalborg University, Aalborg, Denmark; 3 Boehringer Ingelheim Pharma International GmbH, Ingelheim am Rhein, Germany

**Keywords:** atrial fibrillation, oral anticoagulation, patients' perceptions, patient survey, stroke knowledge

## Abstract

**Background** Global differences exist in the management of atrial fibrillation (AF), and cultural differences may influence patients' expectations and perceptions of healthcare. This survey investigated whether country-specific differences in patients' perceptions of AF and oral anticoagulation (OAC) exist and if recent stroke influences these perceptions.

**Methods** Cross-sectional survey of 937 adults with nonvalvular AF receiving OAC for stroke prevention was conducted across United States, Canada, Germany, France, and Japan. Thirty-minute online interviews conducted between April and November 2015 included AF patients with recent stroke, and newly diagnosed AF and established AF, both without recent stroke.

**Results** U.S. patients and those with recent stroke perceived AF as more serious (54.0 and 55.2%, respectively) and were more concerned about stroke (50.0 and 68.0%, respectively). Japanese patients were more likely to perceive AF as not serious (41.0%), but 50.4% were frequently concerned about stroke. Patients in the United States, Canada, and Germany and those without recent stroke preferred to be involved in OAC treatment decisions (either shared decision making or patient's choice), while French and Japanese patients and those with recent stroke preferred their doctor to choose. For all country groups, stroke prevention was the most important factor when choosing OAC.

**Conclusion** In this international cohort of AF patients, country-specific differences exist in patients' perceptions of AF, concerns about stroke, and preference for involvement in OAC treatment decisions; recent experience of stroke significantly influenced patients' values and preferences regarding AF and treatment. Stroke prevention was rated as the most important factor when choosing OAC treatment.

## Introduction

Recent clinical guidelines on the management of atrial fibrillation (AF) advocate inclusion of patients' preferences in treatment decisions. 1 2 3 4 However, global differences exist in the management of AF, and cultural differences may influence patient's expectations and perceptions of healthcare. Most research regarding patient's values and preferences in AF patients has been in relation to antithrombotic therapy. 5 6 7 8 9 10 11 However, patient's perspectives vary from study to study and may be related to actual differences in preferences, the composition of the cohort (patient's age, sex, socioeconomic status, comorbidities), whether patients are already receiving oral anticoagulation (OAC), knowledge of AF and stroke, the way in which risk information is presented, and the manner in which responses are elicited. 8 9 10 12 13

Generally, AF patients have poor understanding about AF and its trajectory, and a paucity of knowledge about the increased risk of stroke and benefits/risks of OAC. 8 9 12 14 15 16 17 Although a recent prospective survey in eight European countries demonstrated that among AF patients taking OAC, 90% knew that OAC was “to thin the blood,” only 26% were aware that OAC increased the risk of all bleeding, including major bleeding. 18 Furthermore, patients' individual circumstances, previous experiences, and current health status will influence their knowledge and help determine their willingness to take treatments. 8 12 19 Indeed, a prospective U.S. survey 12 found that knowledge of stroke was better among AF patients with previous stroke, and stroke survivors were more willing to take OAC.

Studies initiated before the non–vitamin K antagonist oral anticoagulant (NOAC) era have generally demonstrated that patients place greater importance on avoidance of a stroke rather than bleeding and other OAC-related issues (i.e., regular monitoring, drug–food–alcohol interactions). 5 7 11 20 21 Since the introduction of the NOACs, there has been renewed interest in the values and preferences regarding OAC of AF patients. Only a few studies have compared patient's preferences for vitamin K antagonists (VKAs) and NOACs 6 22 23 and examined the attributes that patients perceive as important when choosing OAC. 6 12 22 23 24 Three studies 6 12 23 reported that patients rate stroke prevention as the most important attribute for OAC, while others rate ease of administration 22 and availability of an antidote 24 as of highest importance; however, differences in the methodology of these studies may account for the disparity in the findings. In addition, these studies did not examine patient's perceptions of AF and stroke, or knowledge about stroke, which may drive these preferences.

We performed a prospective, international survey to investigate whether country-specific differences exist in patient's perceptions of AF, concerns about stroke, stroke knowledge, preferences for OAC treatment decisions, self-reported OAC medication adherence, and the attributes of OAC affecting treatment choice. We also examined whether AF patients who experienced a recent stroke differ in their perceptions about AF, stroke, and OAC treatment compared with AF patients who were free from recent stroke.

## Materials and Methods

### Study Design

This was an international, prospective, cross-sectional study of AF patients aged ≥18 years, receiving OAC for stroke prevention. Patients with mechanical valves or scheduled for heart valve surgery were excluded. Patients were recruited from five countries—United States, Canada, Germany, France, and Japan—through consumer panels, physician/nurse referral, and patient associations. These countries were chosen as they already had wide NOAC usage at the time of the survey and were regarded as representative of key regions (North America, South-East Asia, and Europe). Patients were recruited depending on their AF status and stratified into three groups according to predefined quotas: (1) AF with recent stroke (≤6 months, regardless of when AF was diagnosed); (2) newly diagnosed AF without recent stroke (diagnosed with AF ≤6 months [United States, Canada, France, Germany], or ≤12 months in Japan); and (3) established AF without recent stroke (AF diagnosed 7–24 months previously [United States, Canada, France, Germany], or 1–3 years previously in Japan).

Qualitative interviews were performed with 16 AF patients (6 in Germany, 10 in the United States) prior to release of the online survey, to test and optimize the readability of questions and patient information. For the online survey, a total of 517,692 individuals were contacted, 517,203 of those through country-specific consumer panels (6 or 7 different panels per country). A total of 489 were contacted via phone (all countries) or face-to-face (France, Japan) from physician/nurse/pharmacist referral. Referral from patient associations was more common outside of North America; online forums/social media were employed in Germany and Japan. Of 89,481 individuals who connected to the online survey, 7,889 eligible AF patients were identified, of which 937 (11.9%) completed the 30-minute survey in their native language between April and November 2015. The questionnaire consisted of 22 questions to elicit demographic data (age, sex, country/region, highest level of education), past medical history, and the outcomes of interest. All data were collected anonymously and analyzed in an aggregated fashion.

**Fig. 1:**
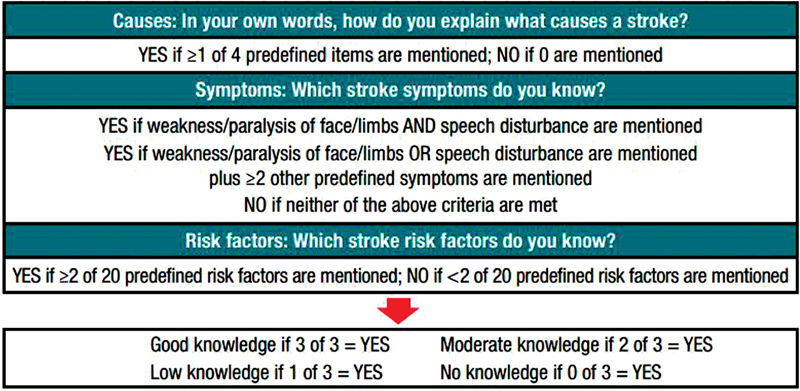
Scoring system to categorize patients' level of stroke knowledge (from open-ended questions).

For the current analyses, the outcomes of interest were patient's perceptions of the seriousness of AF and their concerns about stroke, patient's knowledge about stroke, self-reported adherence to OAC, patient's preference for involvement in OAC treatment choice, and attributes of OAC affecting treatment choice by the patient. Assessment of stroke knowledge is summarized in Fig. 1 . In addition, patients were asked how familiar they were with standardized information about AF and stroke, but this was separate to the assessment of stroke knowledge. The modified Rankin's score (mRS) in patients with a recent stroke was self-reported according to predefined mRS scale descriptors. 25

Sample sizes/quotas were determined according to feasibility estimates and to allow meaningful comparisons across the three predefined AF groups and countries. Participants gave informed consent by completing and submitting the online survey. The survey was conducted in accordance with the principles of the Declaration of Helsinki.

### Statistical Analysis

Descriptive statistics are presented as mean and standard deviation (SD) for normally distributed continuous variables; categorical data are presented as actual count and percentage. Analyses were conducted by country and separately by AF status at baseline (AF with recent stroke vs. AF without recent stroke). To examine differences between countries and between AF groups, means were compared using *t* -tests and categorical data were compared using chi-squared tests. Since heterogeneity was present in the data, we used the unequal variances *t* -test (Welch–Satterthwaite test) to detect differences in means of samples; this test is robust for analyzing equality of means when the homogeneity assumption is not satisfied. Data were analyzed using Sawtooth: Lighthouse Studio v9.0.1 and SPSS PASW Statistics, version 18, with *p-* values <0.05 considered statistically significant. *p* -values comparing more than three subgroups relate to data of one group compared with the data of all other groups together.

## Results

In total, 937 AF patients were recruited with an overall mean (SD) age of 54.3 (16.6) years; 37.1% were female. One-third of patients were from the United States, with between 15 and 18% recruited from Canada, Japan, France, and Germany. Significantly more American women and significantly fewer Japanese women were recruited compared with other countries. Significantly more Americans and Japanese were educated to university/technical college level. Stroke risk was lower among patients from France and Germany and highest among patients from Japan ( Table 1 ).

**Table 1:** Patient baseline demographic and clinical characteristics overall and by country

Characteristic	Overall ( *n* = 937)	USA ( *n* = 322)	Canada ( *n* = 145)	Japan ( *n* = 139)	France ( *n* = 171)	Germany ( *n* = 160)
Mean (SD) age, y	54.3 (16.6)	56.0 (16.9) a	52.8 (17.4)	59.1 (11.8) b	49.6 (18.8) b	53.0 (14.6)
≥65 y	309 (33.0)	128 (39.8) b	45 (31.0)	48 (34.5)	45 (26.3) a	43 (26.9)
Female, *n* (%)	348 (37.1)	151 (46.9) b	55 (37.9)	32 (23.0) b	56 (32.7)	54 (33.8)
Mean (SD) CHA _2_ DS _2_ -VASc score	2.6 (1.7)	2.8 (1.6) b	2.7 (1.5)	3.1 (2.1) b	2.1 (1.7) b	2.3 (1.4) b
CHA _2_ DS _2_ -VASc ≥2 (female); ≥1 (male), *n* (%)	796 (85.0)	280 (87.0)	129 (89.0)	123 (88.5)	130 (76.0) b	134 (83.8)
Educational level, c *n* (%)						
University/technical college	391 (41.8)	178 (55.3) b	55 (37.9)	83 (60.1) b	38 (22.2) b	37 (23.1) b
Community college	263 (28.1)	75 (23.3) a	46 (31.7)	31 (22.5)	55 (32.2)	56 (35.0) a
High-school diploma	255 (27.2)	68 (21.1) b	36 (24.8)	21 (15.2) b	63 (36.8) b	67 (41.9) b
No school leaving certificate	27 (2.9)	1 (0.3) b	8 (5.5) a	3 (2.2)	15 (8.8) b	0 a
AF groups, *n* (%)						
AF with recent stroke	194 (20.7)	63 (19.6)	31 (21.4)	35 (25.2)	34 (19.9)	31 (19.4)
Recent AF, no recent stroke	342 (36.5)	123 (38.2)	50 (34.5)	50 (36.0)	59 (34.5)	60 (37.5)
Established AF, no recent stroke	401 (42.8)	136 (42.2)	64 (44.1)	54 (38.8)	78 (45.6)	69 (43.1)

Abbreviations: AF, atrial fibrillation; SD, standard deviation.

a *p* < 0.05.

b *p* < 0.001 versus other countries pooled.

c One patient did not report educational level.

### Patient's Perceptions about AF, Stroke, and OAC Medication by Country

AF was perceived by American patients as more serious and by French and German patients as less serious, while 41% of Japanese patients perceived AF as not serious ( Table 2 ). There were no differences in stroke knowledge between the five countries ( Table 2 ). American patients were more concerned about stroke, while French patients were less frequently concerned about stroke ( Table 2 ).

**Table 2:** Patients' perceptions about AF and stroke, level of stroke knowledge, self-reported adherence to OAC, and preference for involvement in OAC treatment choice overall and by country

		Overall ( *n* = 937)	USA ( *n* = 322)	Canada ( *n* = 145)	Japan ( *n* = 139)	France ( *n* = 171)	Germany ( *n* = 160)
Perception of seriousness of AF (%)	Extremely/very serious	39.4	54.0 a	40.0	35.3	28.7 a	24.4 a
	Somewhat serious	41.6	33.9 a	47.6	23.7 a	55.0 a	53.1 a
	Not at all/not serious	19.0	12.1 a	12.4 b	41.0 a	16.4	22.5
Concern about stroke (%)	Often/always	43.4	50.0 a	36.6	50.4	32.2 a	42.5
	Occasionally	45.4	41.0	55.2 a	34.5 a	52.6 b	46.9
	Never/don't know	11.2	9.0	8.3	15.1	15.2	10.6
Knowledge of stroke (%)	Good/moderate	47.4	45.3	49.7	48.2	42.7	53.8
	Low/none	52.6	54.7	50.3	51.8	57.3	46.2
Self-reported adherence to OAC	Always take as prescribed	79.9	83.5 b	84.8	67.4 a	80.1	78.8
	Often take as prescribed	17.4	15.2	13.8	26.8 a	18.1	16.2
	Sometimes take as prescribed	2.4	0.9 b	1.4	5.1 b	1.2	5.0 b
	Rarely take as prescribed	0.3	0.3	0	0.7	0.6	0
Patient's preference for involvement in OAC treatment choice	Patient's choice	19.6	19.9	22.1	18.0	14.6	23.8
	Patient–doctor's choice	35.6	41.9 a	37.2	23.7 a	25.7 a	42.5 b
	Doctor's choice	44.7	38.2 a	40.7	58.3 a	59.6 a	33.8 a

Abbreviations: AF, atrial fibrillation; OAC, oral anticoagulation.

a *p* < 0.001 versus other countries pooled.

b *p* < 0.05.

**Fig. 2:**
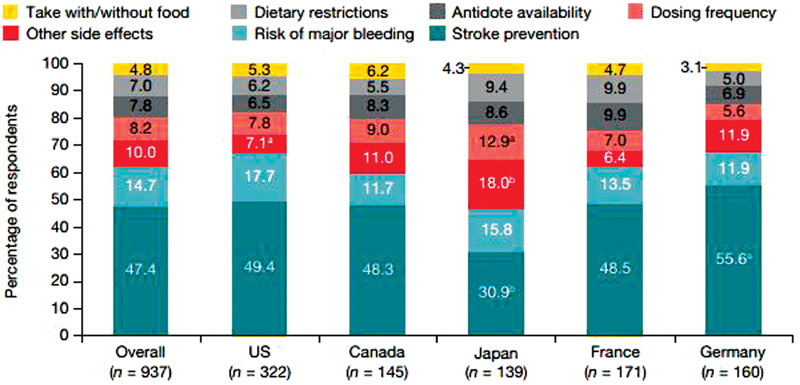
The most important factor in the choice of oral anticoagulation rated by atrial fibrillation patients for the cohort overall and by country. ^a^ *p* < 0.05 versus other countries pooled. ^b^ *p* < 0.001 versus other countries pooled. Percentages do not always equal to 100 due to rounding.

Self-reported OAC adherence was high overall, but American patients reported greater OAC adherence while Japanese patients reported poorer adherence ( Table 2 ). The majority of French and Japanese patients (60 and 58%, respectively) preferred their doctor to choose OAC treatment; in contrast, 62, 59, and 66% of American, Canadian, and German patients, respectively, preferred shared decision making or to choose the OAC themselves ( Table 2 ). Stroke prevention was the most important factor for 47.4% of AF patients when choosing OAC ( Fig. 2 ), followed by risk of major bleeding. Dosing frequency was rated as most important by only 8.2% of the patients. Japanese patients were more concerned about other side effects and dosing frequency than patients from other countries ( Fig. 2 ). Ranking of OAC attributes demonstrated a very similar pattern when analyzed by age (<65 and ≥65 years), sex, or educational level (data not shown).

### Patient's Perceptions about AF, stroke, and OAC Medication among AF Patients with Recent Stroke versus those with No Recent Stroke

Recent stroke occurred in 194 (20.7%) patients; one-third of patients were female. Patients with recent stroke were significantly younger (47.6 [17.5] years vs. 56.0 [15.9] years, respectively) and had a significantly higher CHA _2_ DS _2_ -VASc score (4.3 [1.3] vs. 2.2 [1.5]) than patients without a recent stroke (both *p* < 0.001). Of those with a recent stroke, 149 of 193 (77.2%) had a moderate to severe disability (mRS: 2–5); mean mRS (SD) score was 2.5 (1.2).

Patients with recent stroke were significantly more likely to perceive AF as an extremely serious or very serious condition and were often more concerned about stroke than those without a recent stroke ( Table 3 ). Those with less functional disability after a stroke (mRS score: 0–1) were more likely to perceive AF as not serious compared with those with moderate to severe disability (18.2 vs. 3.4%, respectively). Good levels of stroke knowledge were significantly lower in patients with a recent stroke compared with those without (9.8 vs. 22.1%, respectively, *p* < 0.001). There were high levels of self-reported adherence to OAC therapy, but recent stroke survivors reported significantly lower adherence to OAC compared with patients without a recent stroke. AF patients with a recent stroke preferred their doctor to make the OAC treatment choice, whereas patients without a recent stroke were more likely to prefer being involved in the decision ( Table 3 ).

**Table 3:** Patients' perceptions about AF and stroke, level of stroke knowledge, self-reported adherence to OAC, and preference for involvement in OAC treatment choice overall and by recent stroke/no recent stroke

		Recent stroke ( *n* = 194)	No recent stroke ( *n* = 743)
Perception of seriousness of AF (%)	Extremely/ very serious	55.2 a	35.3
	Somewhat serious	37.6	42.7
	Not at all/ not serious	7.2 a	22.1
Concern about stroke (%)	Often/always	68.0 a	37.0
	Occasionally	28.4 a	49.8
	Never/ don't know	3.6 a	13.2
Knowledge of stroke (%)	Good	9.8 a	22.1
	Moderate	27.3	28.0
	Low	30.4	29.6
	None	32.5 a	20.3
Familiarity with information on AF and stroke (%) b	Familiar	53.1	35.3
Self-reported adherence to OAC	Always take as prescribed	73.6 c	81.6
	Often take as prescribed	21.2	16.4
	Sometimes take as prescribed	4.7 c	1.7
	Rarely take as prescribed	0.5	0.3
Patient's preference for involvement in OAC treatment choice	Patient's choice	22.7	18.8
	Patient–doctor's choice	18.6 a	40.1
	Doctor's choice	58.6 a	41.0

Abbreviations: AF, atrial fibrillation; OAC, oral anticoagulation.

a *p* < 0.001.

b 8–10 on a 10-point scale; see supplement.

c *p* < 0.05.

**Fig. 3:**
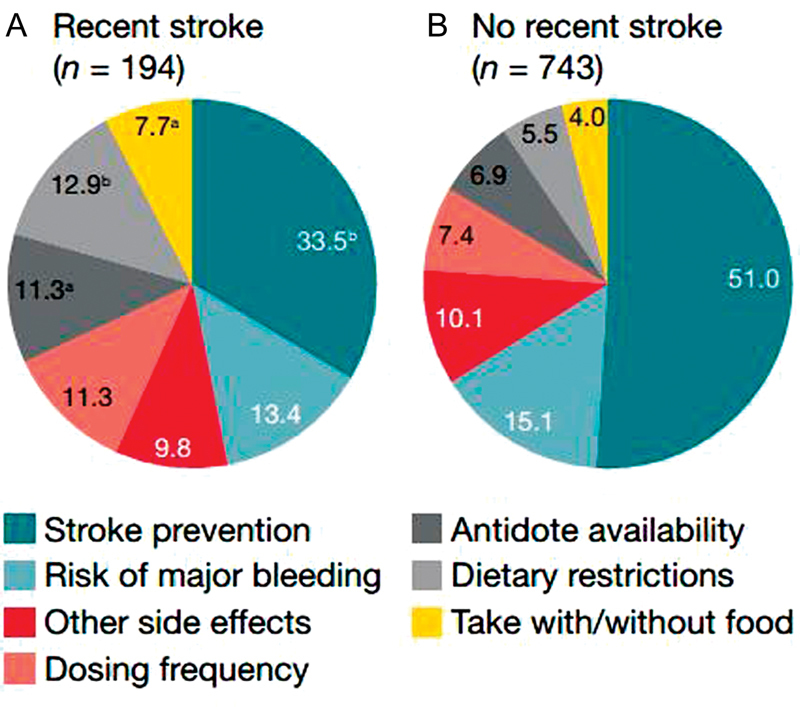
The most important factor in the choice of oral anticoagulation rated by atrial fibrillation patients with a recent stroke ( **A** ) and those without a recent stroke ( **B** ). ^a^ *p* < 0.05 versus no recent stroke. ^b^ *p* < 0.001 versus no recent stroke. Percentages do not always equal to 100 due to rounding.

Stroke prevention was the most important factor for all AF patients when choosing OAC, followed by major bleeding ( Fig. 2 ). AF patients with a recent stroke were less concerned about stroke prevention and more concerned about dietary restrictions, taking medication with/without food, dosing frequency, and antidote availability compared with those without recent stroke ( Fig. 3 ).

## Discussion

In this prospective, international survey of AF patients receiving OAC, there were significant between-country differences in patient's perceptions about AF, stroke, and OAC treatment, and differences between AF patients with and without recent stroke. Concern about stroke was greater among those with recent stroke, while stroke knowledge was poorer. Importantly, AF patients with recent stroke preferred their doctor to make the OAC treatment choice, while patients without recent stroke preferred involvement in this decision making. Finally, stroke prevention was rated as the most important factor when choosing OAC, regardless of country of residence, whether they had experienced a recent stroke, and age, sex, or educational level.

In this survey, country differences in patient's perceptions were evident. American patients perceived AF more seriously and were more concerned about stroke than French patients. Japanese patients were more likely to perceive AF as not serious, but half were highly concerned about stroke. Patients in the United States, Canada, and Germany preferred to be involved in the OAC treatment decision (either shared decision making or patient's choice), while those in France and Japan preferred their doctor to choose. Patients with recent stroke and those with greater functional disability perceived AF as more serious. Country differences may reflect different cultural backgrounds, such as how the physician's role and responsibility is generally perceived in a society, but also specific factors such as educational programs on AF and/or anticoagulation or differences in healthcare systems. Although generic education on AF should be given to all AF patients, our findings suggest that country-specific approaches may be required for certain aspects, such as the discussion about potential consequences of AF to enable patients to understand the seriousness of AF. Importantly, our results suggest that physicians should be aware about the patients' desire to be involved in the OAC treatment decision and act accordingly when talking with the patient as this differs between countries.

Before the initiation of the present study, only a few studies had examined patient's preferences regarding OAC, 7 11 14 predominantly relating to patient's preferences for VKAs 11 as most were conducted before NOACs became available. Given the greater choice of OAC, the differing clinical risk profile (efficacy/safety), differing dosing regimens, and other attributes of the NOACs (reversibility, taking with/without food, etc.)—all of which may affect patient's preference for OAC treatment—this study was advantageous. More recently, there have been a few published studies on patient's preferences for OAC including the NOACs, 6 18 22 23 24 26 but the majority have recruited AF patients from just one country, have much smaller sample sizes, and included more selected AF populations; to our knowledge, the only exception to this was the European Heart Rhythm Association (EHRA) survey. 18 26

The present survey recruited a large sample of AF patients from five countries (three outside Europe), including newly diagnosed AF patients and those with recent experience of stroke, thereby offering a more diverse picture of patient's perceptions of AF and preferences for OAC treatment.

### Perceptions of Atrial Fibrillation and Concerns about Stroke

Previous studies have demonstrated that patients often do not perceive AF as a serious condition and are not aware of the increased risk of stroke associated with AF. 8 9 12 14 15 16 Generally, patients' knowledge about AF and stroke prevention is poor, 8 9 12 15 16 and educational level does not always differentiate those who know the purpose of OAC from those who do not. 18 26 However, the majority of studies report that patients are concerned about the risk of stroke and wish to avoid this. 7 10 20 21 Indeed, a Canadian study 7 elicited health utilities using an iPad questionnaire and found that patients viewed minor stroke as slightly worse than a major bleed, whereas a moderate stroke was viewed as virtually equivalent to death and a major stroke as worse than death. Studies have generally shown that patients are prepared to accept a higher risk of OAC-related bleeding to prevent stroke. 7 10 21 How AF patients are informed about their risk profile and available treatments can affect treatment choice, and this is likely to be highly variable in clinical practice. A recent EHRA survey 27 found that 51% of centers had structured education programs for stroke prevention for AF patients. According to respondents, patient's preferences for OAC were considered important when making treatment decisions in the majority of centers (64.7%). Although some centers have resources/programs in place to educate patients about their stroke risk profile and treatment, there was a disparity in what was delivered and delivery strategies employed. 27

### Participation in the Oral Anticoagulation Decision

In line with the recommended shared decision-making approach, 28 29 30 31 32 it is evident from the present study that a large proportion of AF patients prefer to be involved in the choice regarding OAC (55.3% overall), while many others prefer the physician to choose the OAC. However, country-specific differences and experience of stroke can affect a patient's desire to participate in decision making and may reflect different sociocultural role expectations in the patient–physician interaction; educational level did not influence this preference. One small cross-sectional study reported that 98% would like to participate in the OAC decision; 24 however, several factors, including the small sample size, an all-male U.S. veteran cohort, and a mixture of patients with and without AF—with some of them receiving OAC—limit these findings. A qualitative study 20 of AF patients requiring OAC showed that AF patients prefer consultations that provide the opportunity to make an informed decision, and that they favor an individual approach based on their risk profile (stroke and bleeding) and the attributes that are important to them.

Although some patients may not wish to participate in the treatment decision, at the very least an approach that encourages patient–physician dialogue should be advocated to increase the likelihood of healthcare professionals (HCPs) imparting adequate information to the patient in order for them to understand the condition and the need for OAC treatment and, hence, the potential implications of treatment decisions (advantages and disadvantages). A contemporary EHRA consensus document highlights the importance of education of AF patients 8 and repetition of information over time, from various sources.

### Self-reported Adherence to Oral Anticoagulation

Adherence to OAC medication is essential to minimize thromboembolic or hemorrhagic complications, and physicians are often concerned about patient adherence to OAC. 33 34 35 Overall, self-reported adherence to OAC was high in the present study (79.9%). American patients reported greater adherence to OAC (83.5%), while Japanese patients reported poorer adherence (67.4%), which inversely correlated with the perception of seriousness of AF (41% of Japanese patients believed AF was not serious compared with only 12% of American patients). Patients with recent stroke reported lower adherence, which may be due to cognitive impairment, or reflect limited trust in the efficacy of OAC or vice versa being a cause for the patient to have suffered a (hemorrhagic) stroke. Self-reported adherence to OAC was around 75% in a recent EHRA survey of 1,147 patients with AF from eight European countries; 26 adherence was significantly lower in men versus women and younger versus older patients (<65 vs. ≥65 years). Other studies employing more objective measures of adherence (e.g., proportion of days medication is taken as prescribed based on prescriptions or electronic monitoring devices) 36 37 38 39 demonstrates that NOAC adherence is generally 80% or better, but adherence rates vary between cohorts and by the definition, and measure, of adherence employed.

### Factors Important in Oral Anticoagulation Choice

The present study found that patients place greater importance on avoidance of a stroke than bleeding, and on efficacy over factors associated with treatment burden (e.g., dosing frequency, dietary restrictions, taking the medication with or without food) when ranking attributes of OAC treatment. This finding concurs with most other studies, 6 23 25 40 including a systematic review of patient's values and preferences in decision making for antithrombotic therapy. 11 A study of AF patients with and without stroke demonstrated that reducing the risk of ischemic stroke was the primary factor for OAC treatment choice, followed by the medication with least side effects, then intracranial hemorrhage, then ease of use (e.g., once-daily dosing), and finally cost. 12

A recent Canadian survey 6 of 266 AF patients receiving OAC (warfarin or NOACs) for stroke prevention found that views on importance of OAC attributes differed between patients and physicians. Another multicenter German study of AF patients receiving uninterrupted OAC over the previous 3 months (either VKA or rivaroxaban) 22 employed discrete choice experiment methodology to rate (yes/no) the treatment-related attributes of OAC preferred. Patients preferred OAC treatment that was easy to administer (no bridging, once daily, no food interactions, without international normalized ratio checks/dose adjustment), and less distance to travel to their HCP. However, this study did not include efficacy and safety attributes among the choices, which is problematic given that these factors are likely to affect which attributes the patient perceives as “most important” and may alter treatment preferences. A Swiss prospective, observational, cross-sectional study (PRiSMA-AF [AF Patient Preferences toward NOAC versus VKA Treatment: A Patient Preference Study]) employing the same methodology and attributes as the study by Böttger et al 22 is also examining AF patients' preferences for OAC (VKA and the four NOACs). In a smaller study of U.S. veterans, 24 the most important factors associated with opting for a particular OAC (from most to least important) were availability of an antidote, quality of life, physician recommendation, length of time available in the marketplace, more information before the decision, and lower stroke risk.

## Limitations

All patients in the present study were receiving OAC and therefore their previous experiences (good or bad) with OAC could have influenced their responses. Patients may have wanted to prevent cognitive dissonance (internal mental conflict) and therefore replied so that their responses matched their current medication, or may have given the answer they felt was required rather than their true preference (social desirability bias). The opportunistic sampling strategy based on patients' willingness to complete an online survey may have biased the sample toward being younger (and more technology-savvy), as evident from the mean age of the cohort, and may have influenced the findings and importance of certain attributes. Therefore, the findings may not be representative of older AF patients or those patients not receiving OAC. Also, respondents may be more knowledgeable about AF and OAC treatment than “general” AF patients, and therefore more willing to volunteer to participate in a survey on preferences and knowledge, limiting the generalizability of the results. However, this study recruited a large number of patients from several countries, where differences in the management of AF and cultural differences in the perception of healthcare may affect the outcomes of interest, and therefore the results may be more representative of AF patients globally. Furthermore, preferences for OAC attributes were independent of age, sex, and educational level. In addition, this study included a range of AF patients, those with a recent stroke, newly diagnosed, and established AF, as different experiences of living with AF, OAC treatment exposure, and the adverse consequences of AF (i.e., a recent stroke) will likely affect patient's perceptions and treatment preferences, as demonstrated by other studies. 6 12 22 24

## Conclusion

Country-specific differences exist in AF patients' perceptions of AF, concerns about stroke, and preference for involvement in OAC treatment decisions; recent experience of stroke also significantly influenced patients' perceptions of AF and stroke, and preference for involvement in the OAC decision. With the availability of NOACs, physicians have greater choice for OAC, which differ in terms of their clinical profile (risk/benefit), dosing regimen (once vs. twice daily), and other attributes; however, patient's preferences concerning anticoagulation therapy in this context have only been partly evaluated. Regardless of country of residence or whether they had experienced a recent stroke, all patients rated stroke prevention as the most important factor when choosing OAC treatment. Enhancing physician–patient dialogue is important to educate patients about AF and treatment options and to inform physicians about patients' preferred level of involvement in treatment decisions, as this is likely to increase patient's satisfaction, which may result in improved adherence. The findings of this survey could be used to inform patient–physician communication training and patient education programs.
